# Use of the *KlADH3* promoter for the quantitative production of the murine PDE5A isoforms in the yeast *Kluyveromyces lactis*

**DOI:** 10.1186/s12934-017-0779-5

**Published:** 2017-09-22

**Authors:** Silvia Cardarelli, Mauro Giorgi, Fabio Naro, Francesco Malatesta, Stefano Biagioni, Michele Saliola

**Affiliations:** 1 Department of Biology and Biotechnology “C. Darwin”, Sapienza University of Rome, Piazzale A. Moro 5, 00185 Rome, Italy; 2 Department of Anatomical, Histological, Forensic, and Orthopaedic Sciences, Sapienza University of Rome, Piazzale A. Moro 5, 00185 Rome, Italy; 3 Department of Biochemical Sciences “Rossi Fanelli”, Sapienza University of Rome, Piazzale A. Moro 5, 00185 Rome, Italy

**Keywords:** *Kluyveromyces lactis*, *KlADH3* promoter, Multicopy plasmids, Murine PDE5, cGMP, Sildenafil

## Abstract

**Background:**

Phosphodiesterases (PDE) are a superfamily of enzymes that hydrolyse cyclic nucleotides (cAMP/cGMP), signal molecules in transduction pathways regulating crucial aspects of cell life. PDEs regulate the intensity and duration of the cyclic nucleotides signal modulating the downstream biological effect. Due to this critical role associated with the extensive distribution and multiplicity of isozymes, the 11 mammalian families (PDE1 to PDE11) constitute key therapeutic targets. PDE5, one of these cGMP-specific hydrolysing families, is the molecular target of several well known drugs used to treat erectile dysfunction and pulmonary hypertension. *Kluyveromyces lactis*, one of the few yeasts capable of utilizing lactose, is an attractive host alternative to *Saccharomyces cerevisiae* for heterologous protein production. Here we established *K. lactis* as a powerful host for the quantitative production of the murine PDE5 isoforms.

**Results:**

Using the promoter of the highly expressed *KlADH3* gene, multicopy plasmids were engineered to produce the native and recombinant *Mus musculus* PDE5 in *K. lactis*. Yeast cells produced large amounts of the purified A1, A2 and A3 isoforms displaying K_m_, V_max_ and Sildenafil inhibition values similar to those of the native murine enzymes. PDE5 whose yield was nearly 1 mg/g wet weight biomass for all three isozymes (30 mg/L culture), is well tolerated by *K. lactis* cells without major growth deficiencies and interferences with the endogenous cAMP/cGMP signal transduction pathways.

**Conclusions:**

To our knowledge, this is the first time that the entire PDE5 isozymes family containing both regulatory and catalytic domains has been produced at high levels in a heterologous eukaryotic organism. *K. lactis* has been shown to be a very promising host platform for large scale production of mammalian PDEs for biochemical and structural studies and for the development of new specific PDE inhibitors for therapeutic applications in many pathologies.

## Background

Cyclic nucleotides (cNMP) are the components of evolutionary conserved transduction pathways controlling all aspects of cell life [1] and phosphodiesterases (PDEs) have a central role in the cAMP/cGMP hydrolysis [2, 3]. These enzymes modulate the amplitude and duration of the cAMP/cGMP signal within the cell and ultimately the localization, the composition and the activity of protein kinase A (PKA) and G. To date eleven families of class I PDE isozymes, encoded by 24 distinct genes, have been identified in higher eukaryotes displaying distinct biochemical properties and substrate specificity [4]. PDE5, one of these families containing three isoforms encoded by *Pde5a1*, *Pde5a2* and *Pde5a3*, has a major role as cGMP-dependent regulator of vascular smooth muscle contraction. This activity is the molecular target of several well-known drugs used to treat erectile dysfunction and pulmonary hypertension [5].

In *Saccharomyces cerevisiae* two genes for PDE enzymes that hydrolyse cAMP are present and glucose is the extracellular signal that triggers their expression as well as the cAMP-dependent activation of PKA [6, 7]. Pde1 is a “class II” hydrolysing enzyme with low affinity for cAMP and cGMP [8, 9]. Pde2 has high affinity for cAMP and belongs to class I of the higher eukaryotes superfamily [10]. These two *pde* mutants, together with fission yeast mutants strains have been successfully used as powerful genetic tools for the expression and cloning of mammalian PDE genes. The activities have been biochemically characterized and brilliant yeast genetic selections were developed for the identification of many specific PDE inhibitors [11–14]. However, to our knowledge, no yeast has been engineered for the quantitative production of mammalian PDEs.

*Kluyveromyces lactis* harbours two uncharacterized *PDE* genes closely related to those of *S. cerevisiae* suggesting their involvement in the regulation of common pathways. *K. lactis* is a Crabtree–negative GRAS (Generally Recognized as Safe) yeast in which both respiratory and fermentative metabolisms coexist during its growth on glucose [15]. Extensive metabolic and physiological studies have established *K. lactis* as the fermentative-deficient model equivalent but alternative to the respiratory-deficient *S. cerevisiae petite* mutants [16–23]. Indeed, *K. lactis* has been shown to be an attractive host for heterologous protein production [24], for genetic studies and industrial applications, being one of the few yeast species capable of growing on lactose as a sole carbon source [25–27].

The choice of the promoter is an important element in having an efficient and regulated expression of the gene of interest [24, 28]. In these reviews the authors listed the constitutive/regulated yeast promoters successfully used for the production of recombinant proteins. In this paper, the well-characterized promoter of *KlADH3*, a gene highly induced by all respiratory carbon sources with the exception of ethanol that specifically mediates its repression [29–32], was used for the construction of pKD1-derived expression vectors. pKD1 is a natural plasmid originally isolated from *Kluyveromyces drosophilarum*, but stably maintained in *K. lactis* [33–35]. The use of the *KlADH3* promoter and *K. lactis* for the production of PDE5A isoforms under respiratory growth conditions, has been preferred to the Crabtree–positive *S. cerevisiae* to avoid/reduce the powerful signaling role of cNMP/PDEs, key components of glucose transduction pathways [7]. For the same reason, *KlADH3* has been preferred to commonly used promoters like *LAC4*, *GAL7* and even *KlADH4*, genes partially induced in glucose-containing medium [18, 19, 27, 28].

These expression vectors were used for the quantitative production of the *Mus musculus* PDE5 isoforms. We found that the expression of the *M. musculus Pde5a1, a2* and *a3* variants of the *Pde5a* gene, obtained from a pCDNA3.1-cloned cDNA embrio library [36], was well tolerated by *K. lactis* cells without major growth deficiencies. PDE5A proteins, produced in large amounts, displayed biochemical properties identical to those of the native murine isoforms [37]. This process could potentially be extended for the quantitative production of other higher eukaryotes PDE families.

## Methods

### Strains, media and culture conditions

The *K. lactis* CBS2359 (*MATa*) (http://www.cbs.knaw.nl) strain was used in this work. Cultures were grown under shaking conditions at 28 °C in YP (1% Difco yeast extract, 2% Difco Bacto-peptone) or in minimal medium (6.7 g/L Difco Yeast Nitrogen Base) supplemented by carbon sources at the concentration specified in the text. Geneticin (G418) concentration in selective conditions was 100 μg/mL. *Escherichia coli* strain DH5α was used for the propagation of plasmid DNA. Cultures were grown at 37 °C in LB medium (0.5% Difco yeast extract, 1% Difco tryptone, 0.5% NaCl, supplemented with 100 μg/mL ampicillin).

### Construction of plasmids for the expression of the *Pde5a1, a2 and a3* in *K. lactis*

#### Construction of pα-PDE5A1

This vector contains the DNA coding for the killer toxin-secretion peptide (α-signal) [34] fused in frame with the *M. Musculus Pde5a1* cDNA gene. The α-signal DNA was synthesized as two short single strands complementary sequences containing at the 5′ and 3′ end the *Xba*I and *Sal*I sites (Fwd: ctag atg aat ata ttt tac ata ttt ttg ttt ttg ctg tca ttc gtt caa ggt agg ggt gtg ttt cgt cga g; Rev: tc gac tcg acg aaa cac acc cct acc ttg aac gaa tga cag caa aaa caa aaa tat gta aaa tat att cat), respectively, and then annealed. The 2.6 Kbp *Sal*I–*Sma*I PDE5A1 fragment was purified from p3XFLAG-CMV7 [36]. The two fragments were ligated into the Bluescript KS (Stratagene) plasmid digested with *Xba*I–*Sma*I. Transformation and screening of *E. coli* cells led to the isolation of pKS/αsignal-PDE5A1 containing the α-signal DNA correctly fused at the 5′ end of the *Pde5a1* gene. Finally, the recombinant gene, purified as a 2.7 Kbp *Xba*I-digested fragment from pKS/αsignal-PDE5A1, was cloned in the *Nhe*I-digested site of pYG137/1 to harbour pα-PDE5A1 (Fig. 2b).

pYG137/1 (Fig. 2c) is a derivative of pYG132, a multicopy plasmid previously used for the production and secretion of the recombinant human serum albumin under the control of the *KlADH4* gene promoter [33, 35, 38]. This vector contains the entire pKD1 plasmid, the kanamycin bacterial gene that confers resistance to the G418 antibiotic in yeast, the promoter of *KlADH3* [29–32] and the terminator of the *S. cerevisiae PGK1* gene.

pYG137/1 was also used to construct p3XFlagPDE5A1, p3XFlagPDE5A2 and p3XFlagPDE5A3 which contain the *M. musculus Pde5a1, a2* and *a3* spliced-variants of the *Pde5* gene fused at their 5′ end to the short DNA sequence coding for the 3XFLAG peptide (Sigma), an immunogenic peptide for the affinity purification of PDEs from whole cellular extracts. These recombinant vectors were constructed in two stages from pα-PDE5A1 and the p3XFLAG-CMV7 vectors containing either *Pde5a1, a2* or *a3* in frame with the 3XFLAG coding sequence [36]. To start, pα-PDE5A1 was digested with *Pml*I, ligated and transformed in *E. coli* cells. The screening of transformants allowed the isolation of pYG137/1-PDE5Δ0.8, which contains a deletion of 0.8 Kbp of DNA coding for the α peptide and the first 0.75 Kbp of *Pde5a1* (Fig. 2b). In the second stage, the p3XFLAG-CMV7 plasmids were digested with the enzymes *Eco*ICRI and *Pml*I and the 0.7–0.8 Kbp fragments (containing the 3XFLAG fused to the 5′ end of the three genes, respectively) were recovered from each digestion, purified and cloned in pYG137/1-PDE5Δ0.8 digested with *Pml*I. The screening of *E. coli* transformed cells led to the isolation of p3XFlagPDE5A1, p3XFlagPDE5A2 and p3XFlagPDE5A3 (Fig. 2b).

We also constructed p-PDE5A1 (Fig. 2b) which contains *Pde5a1* under the control of the *KlADH3* promoter. This vector was obtained by cloning the 0.8 Kbp *Pml*I–*Eco*ICRI fragment, containing the 5′ end of *PDE5A1* recovered from pcDNA3.1 [36], in the *Pml*I-digested pYG137/1-PDE5Δ0.8.

### Preparation of cellular extracts and PDE activity assay

*Kluyveromyces lactis* cells harbouring the PDE5A plasmids (Fig. 2b) were grown under shaking conditions at 28 °C for up to 5 days in 5 mL of YP supplemented with glucose (YPD) or glycerol (YPG) at the concentration of 2%. Harvest cells were collected by centrifugation, washed twice with distilled water and resuspended in 300 μL of lysis buffer (20 mM Tris–HCl buffer pH 7.2, 0.2 mM EGTA, 5 mM MgCl2, 5 mM β-mercaptoethanol, 0.1% v/v Triton X-100, 2% v/v antiprotease cocktail (Sigma Aldrich, CA, USA), 1 mM PMSF) and broken with glass beads (∅ 0, 5 mm). The lysate was recovered by centrifugation at 14,000*g* for 30 min at 4 °C, ready for further analyses. The recovered pellets were resuspended in 200 μL of lysis buffer and analysed for PDE activity.

PDE activity was measured at 30 °C with the two-step method described by [39] using [^3^H]cGMP (Perkin Elmer, MA, USA). Aliquots of extracts were incubated in 60 mM HEPES pH 7.2 assay buffer containing 0.1 mM EGTA, 5 mM MgCl_2_, 0.5 mg/mL bovine serum albumin, 30 µg/mL soybean trypsin inhibitor, in a final volume of 0.15 mL (PDE assay buffer). The reaction was started by adding tritiated substrate at a final concentration of 1 μM [^3^H]cGMP and stopped by adding 0.1 M HCl. The Sildenafil used in enzymatic inhibition experiments was a generous gift from Pfizer.

### Quantitative purification of the 3XFlagPDE5A1, A2 and A3 isoforms

100 mL of YPG medium were inoculated with the CBS2359 strain, harbouring the 3XFlagPDE5A plasmids, and grown for 4 days under shaking conditions. All purification steps were carried out at 4 °C. Cells were harvested and broken in 10 mL of lysis buffer, as described above and the lysate centrifuged twice at 20,000*g* for an hour. The supernatant was collected, diluted 20 times in the purification buffer (50 mM HEPES pH 7.5, 75 mM NaCl, 37.5 mM MgCl_2_) and loaded for three times in a chromatography column with an ANTI-FLAG M2 affinity gel (Sigma-Aldrich) equilibrated with the purification buffer. The column was washed with 20 mL purification buffer. The elution of the 3XFlagPde5A proteins was performed by competition with the 3XFLAG peptide (Sigma-Aldrich) according to instructions. The eluted fractions were analyzed by western blotting and for PDE activity.

### SDS-PAGE and western blotting

The purity and integrity of proteins were assessed using SDS-PAGE. Protein samples were boiled for 3 min and subjected to 8% SDS-PAGE and then visualized by 0.1% Coomassie Brilliant Blue R-250 staining. For western blotting analysis, after the electrophoresis, the proteins were transferred to nitrocellulose membranes (Bio-Rad, Hercules, CA, USA). Blots were incubated overnight at 4 °C with rabbit polyclonal anti-PDE5 antibody (1:1000; Santa Cruz Biotechnology). Alkaline phosphatase conjugated anti-rabbit IgG (1:5000; Sigma-Aldrich) was used as a secondary antibody to reveal the immune-complexes. Immunoreactive bands were stained with nitroblue tetrazolium (0.3 mg/mL) in the presence of 5-bromo-4-chloro-3-indolyl-phosphate (0.15 mg/mL).

### In-gel native ADH staining assay and PDE activity in gel slice

Cell extract preparation, native polyacrylamide gel electrophoresis (PAGE), electrophoresis conditions and ADH staining assay were carried out as previously described [29]. PDE activities were recovered from the native gel slices with 100 μL of PDE assay buffer by over night-incubation on ice. The samples were then centrifuged at 14,000*g* for 15 min at 4 °C and aliquots of the eluates assayed for cNMP hydrolysing activity.

### General methods

DNA manipulation, plasmid engineering and other techniques were performed according to standard procedures. Yeast transformation was performed by electroporation with a Biorad Gene-Pulser apparatus. Protein concentration was determined according to the Bradford method [40].

## Results

### Analysis of the KlAdh3 activity by native PAGE ADH staining

**Fig. 1:**
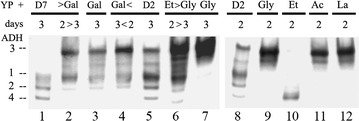
*Kluyveromyces lactis* in-gel native ADH pattern. Cells were grown for 2–3 days in YP medium supplemented with the carbon sources at 2%, unless stated in the text. Extracts from these cultures fractioned on native PAGE were stained for ADH. The migrating positions of ADH isoforms are reported on the left. The  < >  symbols indicate switch of substrate after 2 days of growth

*KlADH3* is regulated at the transcriptional level by carbon sources and a native PAGE ADH staining assay can be used to show its expression [29–32]. To this end, wild type cells were grown for 2–3 days in YP medium containing either fermentative, respiratory or switched to another substrate. Cellular extracts prepared from these cultures were fractioned on native PAGE and stained for ADH activity [29]. As shown in the ADH pattern reported in Fig. 1, KlAdh3 was absent when the cultures were grown in 7% glucose or in ethanol (Fig. 1, lane 1 and 10) [29]. The pattern displayed KlAdh3 when extracts were prepared from cultures containing 2% glucose or galactose (Fig. 1, lanes 3, 5 and 8). The amounts of KlAdh3 were only slightly increased when the cultures were switched from glucose to galactose (Fig. 1, lanes 2 and 4 vs. 3) but these levels were highly increased when cells were switched from ethanol to other respiratory carbon sources like glycerol (lane 10 vs. 6). The extracts displayed the highest amounts of KlAdh3 when the cultures were grown for 2–3 days in YP medium containing either glycerol (Fig. 1, lanes 7 and 9), acetate (lane 11) or lactate (lane 12). In these conditions KlAdh3 was the only Adh present in the pattern (Fig. 1, lanes 7, 9 and 11–12). However, the growth in glycerol, acetate and lactate medium was not identical. In fact, the biomass yield obtained from glycerol, after 3–4 days of growth, was nearly twice the amounts produced in acetate and lactate medium (not shown).

### Engineered plasmids for the production of the murine PDE5A1, A2 and A3 isoforms

**Fig. 2:**
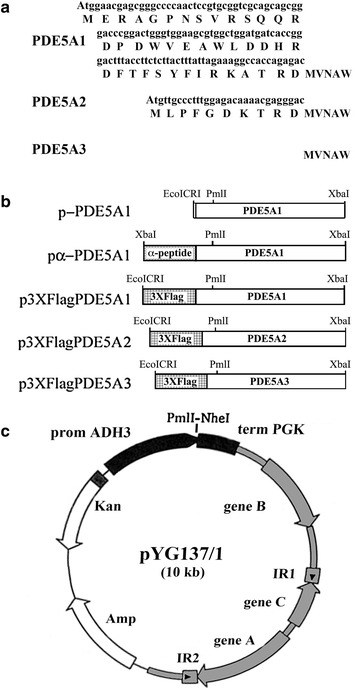
Vectors used for the expression of *Pde5a* genes. **a** N-terminal variants of PDE5A1, A2 and A3 isoforms. **b** Plasmid PDE5A inserts. **c** Map of the pYG137/1 used for the expression of *PDE5A1* and recombinant derivatives. pKD1 sequences in grey. α-peptide/3XFlag schemes are not in scale with those of PDE5A

The reported data suggested to us the use of the *KlADH3* promoter for the construction of an expression cassette. pYG137/1, a multicopy pKD1-derived vector [33], contains this cassette with the *KlADH3* promoter and the *S. cerevisiae PGK1* terminator (Fig. 2c).

The aim of this work was to express *Pde5a1* and secrete its product in the extracellular medium. Therefore, the DNA sequence coding for the killer toxin α-peptide was fused in frame in front of the *Pde5a1* gene. This peptide, engineered for production of human serum albumin, has proved to be an efficient targeting export signal for many heterologous proteins [24, 34, 35, 41]. The final construct, containing the chimeric gene in pYG137/1, was called pα-PDE5A1 (Fig. 2b). pYG137/1 was also used to express the native murine *Pde5a1* and *Pde5a1, a2* and *a3* alternative spliced mRNA variants of the *Pde5* gene only differing in their N-terminal coding products (Fig. 2a) [36] in frame with the 3XFLAG DNA. This sequence encodes an immunogenic peptide used for the affinity purification of the PDE5A isoforms (Fig. 2b). The latter plasmids, called pPDE5A1, p3XFlagPDE5A1, p3XFlagPDE5A2 and p3XFlagPDE5A3, were constructed for the intracellular quantitative production of the native and recombinant PDE5A isoforms (see below).

### Use of the *KlADH3* promoter for the production of PDE5A1

pα-PDE5A1 was introduced by electroporation in the *K. lactis* CBS2359 strain and transformants were grown for up to 5 days in YP medium containing glycerol, a carbon source that induces *KlADH3* [29].

**Fig. 3:**
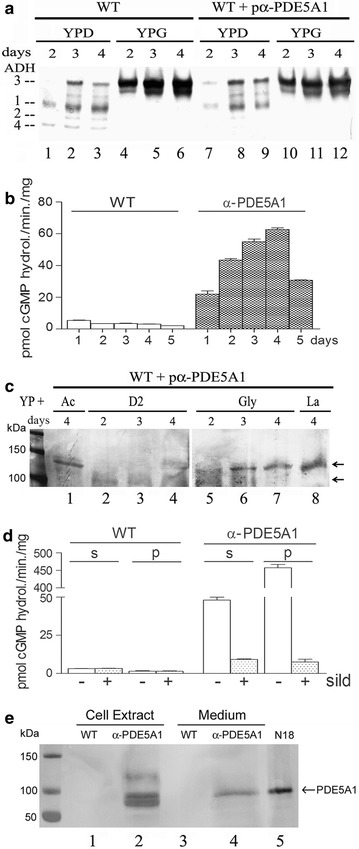
Analysis of ADH and PDE5 activities from cellular extracts. **a** In-gel native ADH pattern from the wild type (WT) and pα-PDE5A1 transformed strain. Cells were grown in YPG or YPD medium for 1–5 days. The migrating position of ADH isoforms is reported on the left. **b** cGMP hydrolysing activities from extracts of WT and pα-PDE5A1 transformed strain. Extracts were obtained from YPG-grown cultures of **a**. **c** Western blot of PDE5A1 from the 2–4 days YPD- YPG-grown cultures extracts of **a** (lanes 7–12) and extracts from YP lactate or acetate cultures. **d** cGMP hydrolysing activities with/without 200 nM Sildenafil (sild). Soluble (s) and pellet fraction (p) were from the 4 days YPG-grown cultures extracts of **a**. **e** Western blot of PDE5A1 from the 4 days YPG-grown cellular extracts and medium supernatants of **a** concentrated 20 times. N18TG2 (N18) murine neuroblastoma cell line extract is used as PDE5 standard. Each lane contains 10 μg of protein extract for ADH analysis, 30 μg for western blot of PDE or the concentrated equivalent of 0.3 mL of medium supernatant. Arrows indicate the migrating position of PDE5 bands

To prove that the *KlADH3* promoter is suited for the production of PDE5A1, cellular extracts, prepared from wild type and transformed strain cultures grown in glycerol and glucose medium, were fractioned on native PAGE and stained for ADH activity. As shown in Fig. 3a, both strains displayed increased amounts of KlAdh3 activity in glycerol conditions (lanes 4–6 and 10–12) as compared with glucose-grown cultures (lanes 1–3 and 7–9). Moreover, cellular extracts prepared from YPG-grown cultures displayed higher amounts of cGMP hydrolysing activities at increased days of growth only in the transformed strain (Fig. 3b) and with a profile that reflects that of KlAdh3 (Fig. 3a, lanes 10–12). In contrast, the wild type only showed very low levels of cGMP-hydrolysing activity independently of the days of growth (Fig. 3b).

The glycerol- and glucose-extracts from the transformed strain (Fig. 3a, lanes 7–12) were also analysed by immuno blot to determine the intracellular accumulation levels of the α-PDE5A1 protein (Fig. 3c). The PDE5 antibody revealed the presence of at least two bands, a faint one of 100 kDa mainly visible at 2 days of growth (Fig. 3c, lane 2 and 5) and a slower migrating band of about 130 kDa accumulated at higher levels in extracts from respiratory carbon source grown-cultures (lanes 1 and 6–8). In contrast, cells grown in YPD showed very low levels of activity (Fig. 3c, lanes 2–4). The lactate-grown culture accumulated higher amounts of putative PDE5A1 than glycerol and acetate-grown ones (Fig. 3c, lane 8 vs. lanes 1 and 7) but, as stated earlier, with a lower biomass yield.

### The α-signal is incapable of promoting the secretion of PDE5A1

To prove that the slower migrating bands visualized in the immuno blot (Fig. 3c, lanes 1 and 6–8) and the cGMP-hydrolysing activities, accumulated by the pα-PDE5A1 transformed strain (Fig. 3b, days 1–5), are PDE5A1, we assayed both soluble and pellet fractions of the extracts in the presence of Sildenafil, the specific inhibitor of PDE5. Indeed, as reported in Fig. 3d, whole extracts showed highly reduced levels of activity in the presence of Sildenafil in both soluble and pellet fractions confirming its identity. However, the amounts of PDE5 measured in the pellet fraction were ten times higher than in the soluble fraction. In contrast, a very small amount of activity was measured in the pellet fraction of the parental untransformed strain and at even lower levels than in the soluble fraction (Fig. 3d).

Unexpectedly, a very small amount of PDE (0.85 pmol cGMP hydrolised/min/mL) was measured in the medium supernatant of the transformed glycerol-grown strain but only after it was concentrated 20 times (Fig. 3e).

To test whether the α-signal is suited for the secretion of PDE5A1 in the medium, the cellular extracts and the medium fractions were concentrated and subjected to immuno blot analysis. The PDE antibody showed the presence of three putative bands of PDE5A1 in the cellular extract (Fig. 3e, lanes 2) indicating that large amounts of the chimeric protein are blocked inside the cell and unable to be secreted in the medium supernatant. In fact, only one of these bands corresponds to the faint one detected in the concentrated growth medium and to the PDE5A1 control band fractioned from the N18TG2 cells extract (Fig. 3e, lanes 4 and 5). N18TG2 is a murine neuroblastoma cell line with very high levels of PDE5A1 [42, 43].

**Fig. 4:**
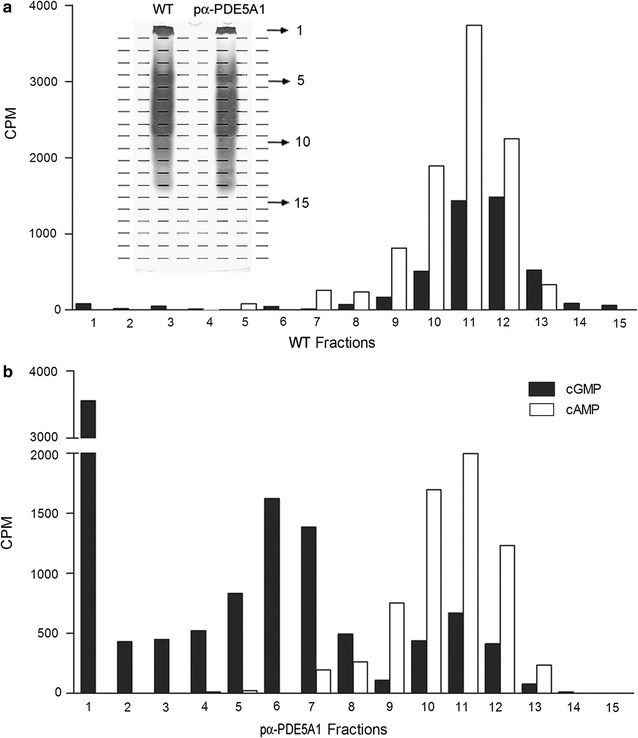
Native PAGE-migrating properties of cAMP/cGMP hydrolysing activities from WT and pα-PDE5A1 culture extracts. The activities of both extracts were determined in CPM (counts per minute) in the sequentially-sliced (1–20) migrated extracts shown in the Coomassie-stained gel insert. Extracts were obtained from cells grown in YPG medium for 4 days

To determine the migrating properties of the endogenous *K. lactis* PDE activities with those of the heterologous PDE5A1, we performed a native PAGE analysis of the cellular extracts from the transformed and wild type strain cultures. Although a native staining method to reveal the PDE activities exists [44], we were unable to visualize bands of PDE with this method (not shown). To overcome this problem, we cut the loaded gel lanes in 20 consecutive slices (three mm large), elute the proteins and test each sample for cAMP and cGMP hydrolysing activities. Slices from wild type extract showed coincidental cNMP hydrolysing activities mainly concentrated in the fractions 10–12 (Fig. 4, upper panel). Although the role of cGMP as second messenger in the metabolism of lower eukaryotes has not been characterized, *K. lactis* like *S. cerevisiae* [9] harbours PDE activities able to hydrolyse both cNMP. The transformed strain expressed, beside the endogenous PDEs (Fig. 4 lower panel, fractions 10–12), other additional and specific slower migrating peaks of cGMP-hydrolysing activities (fractions 5–7). Moreover, this strain displayed, another huge peak of PDE in the first slice of the gel, activities probably associated with an aggregate of proteins unable to enter and migrate in the gel field. The great amount of activity present in this sample (Fig. 4 lower panel, fraction 1) confirmed that most of the PDE activities remained probably entrapped in the membranes.

### *Kluyveromyces lactis* produces high levels of native PDE5A1 and recombinant 3XFlagPDE5A1 activities

**Fig. 5:**
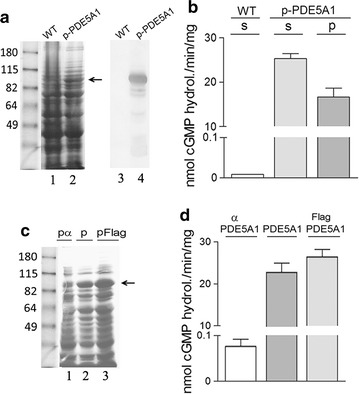
Analyses of PDE5 activities from cellular extracts. **a** SDS-PAGE analysis by Coomassie-staining and western blot of extracts from the WT and the pPDE5A1 transformed strain. **b** cGMP hydrolysing activities from the soluble (s) and pellet fraction (p) extracts of **a**. **c** SDS-PAGE analysis by Coomassie-staining of extracts from the pα-PDE5A1, the pPDE5A1 and the p3XFlagPDE5A1 transformed strains. **d** Determination of cGMP hydrolysing activities from the extracts of **c**. Cultures were grown in YPG medium for 4 days. Each lane contains 10 μg of protein extract. The arrows indicate the bands of PDE5A1

The cells’ inability to secrete PDE5A1 into the medium led us to quantify the intracellular PDE5 accumulated by the p-PDE5A1 and p3XFlagPDE5A1 transformed strains (Fig. 2b). The cellular extract from the glycerol-grown p-PDE5A1 harbouring strain, once fractioned in SDS-PAGE and stained with Coomassie, showed a large band of the expected molecular weight (MW) of PDE5A1 (Fig. 5a, lane 2), a band absent in the control untransformed strain (lane 1). This band was confirmed to be the murine protein by immunoblot analysis (Fig. 5a, lane 4) and its activity was determined in the intracellular soluble fraction (Fig. 5b). Indeed, differently from the strain harbouring pα-PDE5A1 (Fig. 3d), the amounts of PDE determined in the pellet of the p-PDE5A1 strain were only a portion of the amounts found in the soluble fraction (Fig. 5b).

Finally, we compared the amounts of PDE5A1 produced in the strains transformed with either pα-PDE5A1, p-PDE5A1 or with p3XFlagPDE5A1. As shown by the arrow in the Coomassie-stained SDS-PAGE (Fig. 5c, lanes 1–3) and by the histograms (Fig. 5d), the activities determined in the intracellular soluble fractions of the three strains suggested that p-PDE5A1 and p3XFlagPDE5A1 are good vectors for the quantitative production of the PDE5A1 isoform. In contrast, the pα-PDE5A1 strain was confirmed to be unable to accumulate significant amounts of intracellular PDE5A1 (Fig. 5d).

### Growth curves and viability of pα-PDE5A1 and p3XFlagPDE5A1 transformed strains

**Fig. 6:**
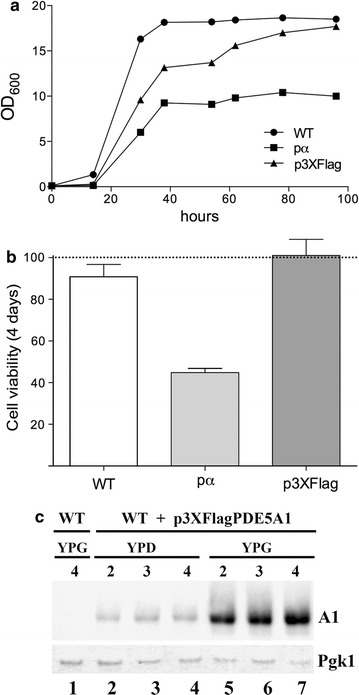
Growth curves and viability of PDE5-transformed strains. **a** Growth curve of WT, pα-PDE5A1 and p3XFlagPDE5A1 transformed strains. Cultures were grown in YPG medium for 4 days and OD_600_ determined at time intervals. Each value is the average of three independent determinations with a standard deviation comprised between 4 and 13%. **b** Cell viability of cultures of A was determined at the end of 4 days of growth. Cell cultures were diluted to 10^8^ cell/mL and the viability expressed as % of those forming colonies on YPD plates. **c** Western blot of PDE5A1 from the 2–4 days YPD-YPG-grown cultures extracts of the p3XFlagPDE5A1 strain. Each lane contains 4 μg of proteins. *S. cerevisiae* Pgk1 antibody was used as loading standard

The production of heterologous proteins is a stress condition that triggers a large common adaptive response, which in yeast is called environmental stress response [24, 45]. Therefore, the 3XFlagPde5A1-producing culture was compared with the one producing α-PDE5A1 that accumulating in the larger part in the insoluble pellet fraction could be subjected to stress. Cell growth at time intervals and cell viability at the end of the 4 days of growth were measured. As shown in Fig. 6a, both transformed strains displayed reduced growths in lag-exponential phases that were recovered to nearly wild type level in the p3XFlagPDE5A1 strain at 4 days of growth. In contrast, the strain transformed with pα-PDE5A1 grew at half the level of the parental strain and reached a precocious stationary phase after 36 h of growth (Fig. 6a). This data confirmed that the secretion signal in front of PDE5A1 had a negative effect on the growth of the transformed cells. Indeed, as shown in Fig. 6b, the viability of the cells, determined as the % of cells capable of forming colonies on plate (cfu) showed in the pα-PDE5A1-containing culture a viability reduced to 50% of that of the other two strains.

The strains transformed with 3XFlagPde5A2 and 3XFlagPde5A3 showed growth curves and viabilities almost identical to that of the p3XFlagPDE5A1 strain (not shown). These results confirmed that overexpression of the murine genes in *K. lactis* leads to a slower adaptation of the cultures, as compared to the parental untransformed strain (Fig. 6a).

Finally, extracts prepared from the p3XFlagPDE5A1-transformed strain, grown for 2–4 days in rich glucose or glycerol medium, were compared for the production of the 3XFlagPDE5A1 isoform by immuno blot using a PDE5 antibody. As shown in Fig. 6c, a small amount of 3XFlagPDE5A1 was visible in the extracts from glucose-grown cells and these levels were independent from the days of growth (lanes 2–4). On the contrary, high levels of 3XFlagPDE5A1 were produced in glycerol-containing medium and greatly increased at 3 and 4 days of growth (Fig. 6c, lanes 5–7). The amounts of 3XFlagPDE5A1 accumulated in the two conditions coincide with the levels of KlAdh3 produced in the same conditions in the parental and transformed strains (Fig. 3a).

### Production and purification of the 3XFlagPDE5 isoforms

**Fig. 7:**
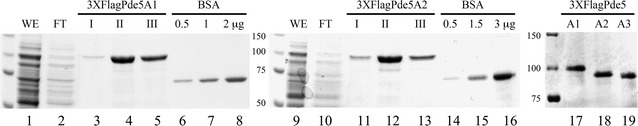
Coomassie-stained SDS-PAGE analysis of the 3XFlagPDE5 isoforms purification processes. The figure reports the whole protein extracts (WE), the flow-through (FT) and the eluted fractions (I, II and III) of the A1 (lanes 1–8) and A2 (lanes 9–16) isoforms purified by an ANTI-FLAG M2 affinity chromatography column. The figure also reports the different MW of the three purified isoforms (lanes 17–19). WE was from 100 mL of YPG cultures grown for 4 days. BSA standards were used for the quantification

The strain harbouring the p3XFlagPDE5A1 construct was used for the quantitative production of the protein. To this end 100 mL of culture was grown under shaking conditions for 4 days, harvested cells were then lysated and the soluble fraction recovered by centrifugation. The 3XFlagPDE5A1 protein was purified from the total extract using an ANTI-FLAG M2 affinity chromatography column and eluted by competition with the 3XFLAG peptide. The purification steps with the whole extract, the flow-through, the eluted fractions and the known amounts of BSA, used to quantify the 3XFlagPDE5A1 protein, are shown in the Coomassie-stained SDS-PAGE of Fig. 7 (lanes 1–8). Identical results were obtained for the quantitative production of the 3XFlagPDE5A2 (Fig. 7, lanes 9–16) and the 3XFlagPDE5A3 (not shown). The MW of the three purified isoforms is shown in the Coomassie-stained SDS-PAGE of Fig. 7 (lanes 17–19). The amounts of the three purified isoforms, obtained from the eluted fractions of 100 mL of YPG culture, were estimated to be between 2.75 and 2.96 mg corresponding to a yield of nearly 30 μg of protein for mL of culture.

**Fig. 8:**
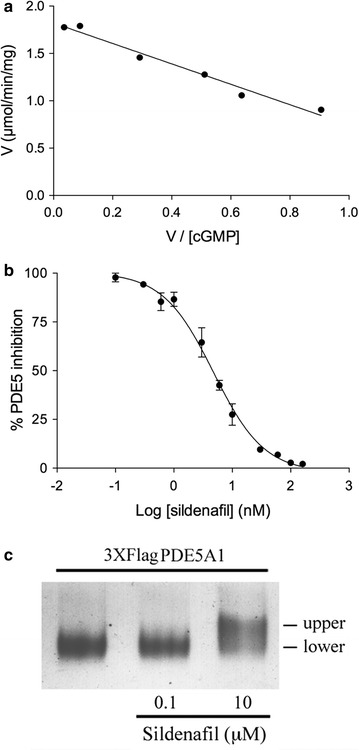
Biochemical properties of the purified 3XFlagPDE5A1 protein. **a** Eadie-Hofstee representation plot of cGMP hydrolysis. **b** Sildenafil inhibition curve. **c** Native PAGE gel shift by pre-incubation of 3XFlagPDE5A1 with Sildenafil. The purified 3XFlagPDE5A1 protein (1 μg) was incubated for 15′ at 30 °C with the inhibitor, fractioned on gel and stained with Coomassie

The purified 3XFlagPDE5A1 was analyzed for kinetic and inhibition properties. In Fig. 8a is reported the Eadie–Hofstee graphical representation of PDE5A1 kinetic with a calculated Km of 1.08 ± 0.08 μM and V_max_ of 1.82 ± 0.04 μmol/min/mg, values not significantly different from those measured in human activity [37]. The inhibitory effect of Sildenafil on PDE5A1 activity, determined using the nonlinear regression analysis of the sigmoidal dose response (Fig. 8b), gave an IC_50_ of 4.7 ± 1.1 nM, a value very similar to that obtained from mouse neuroblastoma immunoprecipitates [46] and human recombinant isoform [47]. Moreover, pre-incubation of the 3XFlagPDE5A1 protein with Sildenafil displayed in native Coomassie-stained PAGE an electrophoretic mobility shift determined by the bond of the inhibitor with the catalytic site of the protein (Fig. 8c) [48]. Biochemical analysis and inhibition potency of Sildenafil determined on PDE5A2 and A3 purified isoforms gave identical results (not shown).

## Discussion

PDEs are cyclic nucleotides hydrolysing enzymes grouped into two classes of proteins with distinct/dual substrate hydrolysing specificity for cAMP and cGMP. Class I groups together more than hundred higher eukaryote isoforms into eleven PDE families (PDE1 to PDE11) [4]. PDE5, one of these families, has three isoforms (A1, A2 and A3) (Fig. 2a) encoded by the *a1*, *a2* and *a3* alternative-spliced variants of the *Pde5a* gene. PDE5 has a major role as cGMP-dependent regulator of vascular smooth muscle contractions and it has been identified as the target of several well-known drugs [5]. The large profit generated by the commercialisation of these drugs led their exploitation for other pathologies expanding the study of PDE5 to other organs. Recently, PDE5 has been shown to be involved in learning/memory processes, in heart failure, in cardio-vascular diseases and in human breast and thyroid cancers [49–53]. Despite the great commercial relevance of these drugs, productions of large amounts of PDE5 isoforms for the development of new specific inhibitors or for the crystal structure determination of the entire PDE5 proteins have either not been carried out or if so, not published. At the moment, crystallographic models have only been constructed for the single catalytic/regulatory domains [54].

In this paper, we showed that the α-peptide signal, in contrast with previous proteins-secretion reports, is unable to target PDE5A1 in the extracellular space (Fig. 3e). In fact, native PAGE, immuno blot analysis and PDE determinations from the protein extracts of the pα-PDE5A1 strain showed that a large amount of the enzyme was located in the insoluble pellet fraction (Figs. 3d, 4 fraction 1) and only a very small amount of PDE5 was detected in the medium (Fig. 3e, lane 4). Moreover, the intracellular accumulated activity displayed PDE5 bands with MWs that vary according to dilutions and/or concentrations of the protein extract (Fig. 3c, e, lane 2). In addition, the pα-PDE5A1 strain enters into a stationary phase at only 36 h of growth showing a reduced vitality, as compared to the other strains (Fig. 6a, b). Since PDEs are components of intracellular signaling pathways, we speculate that the bands of Fig. 3c–e and the activity of the pellet fraction accumulate as unprocessed α-PDE5A1 suggesting the presence of domains conflicting with the secretory apparatus.

On the contrary, the expression cassettes lacking the α-signal allowed the intracellular accumulation of large amounts of the PDE5A1, A2 and A3 isoforms (Figs. 6c, 7). PDE5 yields, nearly 1 mg/g wet weight biomass (30 mg/L culture), are well tolerated by *K. lactis* cells without major growth deficiencies and interferences with the endogenous cAMP/cGMP signal transduction pathways.

These results were made possible by the choice of the host organism, by the use of the *KlADH3* promoter expression vectors and by the growth conditions. *K. lactis* is a Crabtree-negative yeast in which both respiratory and fermentative metabolisms coexist during its growth on glucose. Respiratory growth could be an ideal neutral condition to produce PDE5 isoforms avoiding interferences with glucose transduction pathways [6, 7]. Conversely, *KlADH3* is a gene highly induced during the growth under respiratory growth conditions and pYG137/1 is a multicopy natural pKD1-derived vector. Finally, *K. lactis* transformed strains grow to high yields in rich glycerol medium with only a slightly delayed growth up to early stationary phase (Fig. 6a). A delay probably due to the gradual adaptation of the cells to the synthesis of PDE5A1 accumulating at constant rates during growth phases (Figs. 3b, 6c).

In prospective, the large amounts of the three purified proteins produced could help to define their macromolecular structure by small-angle X-ray scattering (Saxs)/crystallographic studies, to compare the differences between the three isoforms with known structures of other mammalian PDEs [54–56] and for the development of new inhibitors for clinical and therapeutic applications.

## Conclusions

In this paper we report the *KlADH3* promoter-dependent production of the *M. musculus* PDE5A isoforms in *K. lactis*. The use of this promoter was the appropriate choice for the production of PDE5 isoforms as it proved to be well tolerated by *K. lactis* cells. To our knowledge this is the first time that the entire family of PDE5 isoforms containing both regulatory and catalytic domains has been produced in a heterologous eukaryotic organism. The three purified proteins will be used to define their macromolecular structure by crystallographic studies and to compare putative differences between the three isoforms and other mammalian PDEs. These results established *K. lactis* as a model organism for production of mammalian PDE and, in prospective, as a platform to increase their yields by the control of the growth parameters, the use of alternative carbon sources/media and the genetic manipulation of the host.

## References

[1] Weeks G, Spiegelman GB (2003). Roles played by Ras sub family proteins in the cell and developmental biology of microorganisms. Cell Signal.

[2] Omori K, Kotera J (2007). Overview of PDEs and their regulation. Circ Res.

[3] Keravis T, Lugnier C (2012). Cyclic nucleotide phosphodiesterase (PDE) isozymes as targets of the intracellular signalling network: benefits of PDE inhibitors in various diseases and perspectives for future therapeutic development. Br J Pharmacol.

[4] Fajardo AM, Piazza GA, Tinsley HN (2014). The role of cyclic nucleotide signaling pathways in cancer: targets for prevention and treatment. Cancers.

[5] Ioakeimidis N, Kostos JB (2014). Pharmacologic therapy for erectile dysfunction and its interaction with the cardiovascular system. J Cardiovasc Pharmacol Ther.

[6] Santangelo GM (2006). Glucose signalling in *Saccharomyces cerevisiae*. Microbiol Mol Biol Rev.

[7] Conrad M, Schothorst J, Kankipati HG, Van Zeebroeck G, Rubio-Texeira M, Thevelein JM (2014). Nutrient sensing and signaling in the yeast *Saccharomyces cerevisiae*. FEMS Microbiol Rev.

[8] Nikawa J, Sass P, Wigler M (1987). Cloning and characterization of the low-affinity cyclic AMP phosphodiesterase gene of *Saccharomyces cerevisiae*. Mol Cell Biol.

[9] Tian Y, Cui W, Huang M, Robinson H, Wan Y, Wang Y, Ke H (2014). Dual specificity and novel structural folding of yeast phosphodiesterase-1 for hydrolysis of second messengers cyclic adenosine and guanosine 3′,5′-monophosphate. Biochemistry.

[10] Sass P, Field J, Nikawa J, Toda T, Wigler M (1986). Cloning and characterization of the high-affinity cAMP phosphodiesterase of *Saccharomyces cerevisiae*. Proc Natl Acad Sci USA.

[11] Wilson M, Sullivan M, Brown N, Houslay MD (1994). Purification, characterization and analysis of rolipram inhibition of a human type-IVA cyclic AMP-specific phosphodiesterase expressed in yeast. Biochem J.

[12] Cheung PP, Xu H, McLaughlin MM, Ghazaleh FA, Livi GP, Colman RW (1996). Human platelet cGI-PDE: expression in yeast and localization of the catalytic domain by deletion mutagenesis. Blood.

[13] Yu J, Wolda SL, Frazier ALB, Florio VA, Martins TJ, Snyder PB, Harris EAS, McCaw KN, Farrell CA, Steiner B, Bentley JK, Beavo JA, Ferguson K, Gelinas R (1997). Identification and characterisation of a human calmodulin-stimulated phosphodiesterase PDE1B1. Cell Signal.

[14] de Medeiros AS, Hoffman CS (2015). A yeast-based high-throughput screen for modulators of phosphodiesterase activity. Methods Mol Biol.

[15] De Deken RH (1966). The Crabtree effect: a regulatory system in yeast. J Gen Microbiol.

[16] Goffrini P, Algeri AA, Donnini C, Wésolowski-Louvel M, Ferrero I (1989). RAG1 and RAG2: nuclear genes involved in the dependence/independence on mitochondrial respiratory function for the growth on sugars. Yeast.

[17] Wésolowski-Louvel M, Prior C, Bornecque D, Fukuhara H (1992). Rag^−^ mutations involved in glucose metabolism in yeast: isolation and genetic characterization. Yeast.

[18] Schaffrath R, Breunig KD (2002). Genetics and molecular physiology of the yeast *Kluyveromyces lactis*. Fungal Genet Biol.

[19] González Siso MI, Garcia Leiro A, Tarrio N, Cerdán ME (2009). Sugar metabolism, redox balance and oxidative stress response in the respiratory yeast *Kluyveromyces lactis*. Microb Cell Fact.

[20] Cialfi S, Uccelletti D, Carducci A, Wésolowski-Louvel M, Mancini P, Heipieper HJ, Saliola M (2011). KlHsl1 is a component of glycerol response pathways in the milk yeast *Kluyveromyces lactis*. Microbiology.

[21] Saliola M, Tramonti A, Lanini C, Cialfi S, De Biase D, Falcone C (2012). Intracellular NADPH levels affect the oligomeric state of the glucose 6-phosphate dehydrogenase. Eukaryot Cell.

[22] Gorietti D, Zanni E, Palleschi C, Delfini M, Uccelletti D, Saliola M, Miccheli A (2014). Depletion of the unique casein kinase I leads to a NAD(P)+/NAD(P)H balance-dependent metabolic adaptation as determined by NMR spectroscopy-metabolomic profile in *Kluyveromyces lactis*. Biochim Biophys Acta.

[23] Tramonti A, Saliola M (2015). Glucose 6-phosphate and alcohol dehydrogenase activities are components of dynamic macromolecular depots structures. Biochim Biophys Acta.

[24] van Ooyen AJ, Dekker P, Huang M, Olsthoorn MM, Jacobs DI, Colussi PA, Taron CH (2006). Heterologous protein production in the yeast *Kluyveromyces lactis*. FEMS Yeast Res.

[25] Bolotin-Fukuhara M, Rosa C, Peter G (2006). Genomics and biodiversity in yeasts. Biodivers ecophysiol yeasts ser yeast handb.

[26] Koivistoinen OM, Kuivanen J, Barth D, Turkia H, Pitkänen JP, Penttilä M, Richard P (2013). Glycolic acid production in the engineered yeasts *Saccharomyces cerevisiae* and *Kluyveromyces lactis*. Microb Cell Fact.

[27] Rodicio R, Heinisch JJ (2013). Yeast on the milky way: genetics, physiology and biotechnology of *Kluyveromyces lactis*. Yeast.

[28] Weinhandl K, Winkler M, Glieder A, Camattari A (2014). Carbon source dependent promoters in yeasts. Microb Cell Fact.

[29] Saliola M, Falcone C (1995). Two mitochondrial alcohol dehydrogenase activities of *Kluyveromyces lactis* are differentially expressed during respiration and fermentation. Mol Gen Genet.

[30] Saliola M, De Maria I, Lodi T, Fiori A, Falcone C (2006). KlADH3, a gene encoding a mitochondrial alcohol dehydrogenase affects respiratory metabolism and cytochrome content in *Kluyveromyces lactis*. FEMS Yeast Res.

[31] Saliola M, Getuli C, Mazzoni C, Fantozzi I, Falcone C (2007). A new regulatory element mediates ethanol repression of KlADH3, a *Kluyveromyces lactis* gene coding for a mitochondrial alcohol dehydrogenase. FEMS Yeast Res.

[32] Cardarelli S, D’Amici S, Tassone P, Tramonti A, Uccelletti D, Mancini P, Mancini M (2016). Characterization of the transcription factor encoding gene, KlADR1: metabolic role in *Kluyveromyces lactis* and expression in *Saccharomyces cerevisiae*. Microbiology.

[33] Falcone C, Saliola M, Chen XJ, Frontali L, Fukuhara H (1986). Analysis of a 1.6 μm circular plasmid pKD1 from the yeast *Kluyveromyces drosophilarum*: structure and molecular dimorphism. Plasmid.

[34] Fleer R, Chen XJ, Amellal N, Yeh P, Fournier A (1991). High-level secretion of correctly processed recombinant human interleukin-1 beta in *Kluyveromyces lactis*. Gene.

[35] Saliola M, Mazzoni C, Solimando N, Crisà A, Falcone C, Jung G, Fleer R (1999). Use of the KlADH4 promoter for ethanol dependent production of recombinant human serum albumin in *Kluyveromyces lactis*. Appl Environ Microbiol.

[36] Campolo F, Zevini A, Cardarelli S, Monaco L, Barbagallo F, Pellegrini M, Cornacchione M, Di Grazia A, De Arcangelis V, Gianfrilli D, Giorgi M, Lenzi A, Isidori AM, Naro F (2017). Identification of murine phosphodiesterase 5A isoforms and their functional characterization in HL-1 cardiac cell line. J Cell Physiol.

[37] Bender AT, Beavo JA (2006). Cyclic nucleotide phosphodiesterases: molecular regulation to clinical use. Pharmacol Rev.

[38] Falcone C, Fleer R, Saliola M. Yeast promoter and its use (US5627046). US Patent Office. 1997. p. 1–28.

[39] Thompson WJ, Appleman MM (1971). Multiple cyclic nucleotide phosphodiesterase activities from rat brain. Biochemistry.

[40] Bradford MM (1976). A rapid and sensitive method for the quantitation of microgram quantities of protein utilizing the principle of protein-dye binding. Anal Biochem.

[41] Becerra M, Prado SD, Siso MI, Cerdán ME (2001). New secretory strategies for *Kluyveromyces lactis* beta-galactosidase. Protein Eng.

[42] Giorgi M, Caniglia C, Scarsella G, Augusti-Tocco G (1993). Characterization of 3′:5′ cyclic nucleotide phosphodiesterase activities of mouse neuroblastoma N18TG2 cells. FEBS Lett.

[43] Giordano D, Giorgi M, Sette C, Biagioni S, Augusti-Tocco G (1999). cAMP-dependent induction of PDE5 expression in murine neuroblastoma cell differentiation. FEBS Lett.

[44] Goren EN, Hirsch AH, Rosen OM (1971). Activity staining for the detection of cyclic nucleotide phosphodiesterase separated by polyacrylamide gel electrophoresis and its application to the cyclic nucleotide phosphodiesterase of beef heart. Anal Biochem.

[45] Lackner DH, Schmidt MW, Wu S, Wolf DA, Bähler J (2012). Regulation of transcriptome, translation, and proteome in response to environmental stress in fission yeast. Genome Biol.

[46] Giordano D, De Stefano ME, Citro G, Modica A, Giorgi M (2001). Expression of cGMP-binding cGMP-specific phosphodiesterase (PDE5) in mouse tissues and cell lines using an antibody against the enzyme amino-terminal domain. Biochim Biophys Acta.

[47] Corbin JD, Francis SH, Zoraghi R (2006). Tyrosine-612 in PDE5 contributes to higher affinity for vardenafil over sildenafil. Int J Impot Res.

[48] Corbin JD, Zoraghi R, Francis SH (2009). Alloesteric-site and catalytic-site ligand effect on PDE5 functions are associated with distinct changes in physical form of the enzyme. Cell Signal.

[49] Prickaerts J, Sik A, van Staveren WC, Koopmans G, Steinbusch HW (2004). Phosphodiesterase type 5 inhibition improves early memory consolidation of object information. Neurochem Int.

[50] Tsai EJ, Kass DA (2009). Cyclic GMP signaling in cardiovascular pathophysiology and therapeutics. Pharmacol Ther.

[51] Giorgi M, Pompili A, Cardarelli S, Castelli V, Biagioni S, Sancesario G, Gasbarri A (2015). Zaprinast impairs spatial memory by increasing PDE5 expression in the rat hippocampus. Behav Brain Res.

[52] Catalano S, Campana A, Giordano C, Győrffy B, Tarallo R, Rinaldi A, Bruno G, Ferraro A, Romeo F, Lanzino M, Naro F, Bonofiglio D, Andò S, Barone I (2015). Expression and function of phosphodiesterase type 5 in human breast cancer cell lines and tissues: implications for targeted therapy. Clin Cancer Res.

[53] Sponziello M, Verrienti A, Rosignolo F, De Rose RF, Pecce V, Maggiisano V, Durante C, Bullotta S, Damante G, Giacomelli L, Di Gioia CRT, Filetti S, Russo D, Celano M (2015). PDE5 expression in human thyroid tumors and effects of PDE5 inhibitors on growth and migration of cancer cells. Endocrine.

[54] Francis SH, Blount MA, Corbin JD (2011). Mammalian cyclic nucleotide phosphodiesterases: molecular mechanisms and physiological functions. Physiol Rev.

[55] Azevedo MF, Faucz FR, Bimpaki E, Horvath A, Levy I, de Alexandre RB, Ahmad F, Manganiello V, Stratakis CA (2014). Clinical and molecular genetics of the phosphodiesterases (PDEs). Endocr Rev.

[56] Sung BJ, Hwang KY, Jeon YH, Lee JI, Heo YS, Kim JH, Moon J, Yoon JM, Hyun YL, Kim E, Eum SJ, Park SY, Lee JO, Lee TG, Ro S, Cho JM (2003). Structure of the catalytic domain of human phosphodiesterase 5 with bound drug molecole. Nature.

